# Autism in adult and juvenile delinquents: a literature review

**DOI:** 10.1186/s13034-017-0181-4

**Published:** 2017-09-22

**Authors:** A. X. Rutten, R. R. J. M. Vermeiren, Ch. Van Nieuwenhuizen

**Affiliations:** 1 Center for Child & Adolescent Psychiatry, GGzE, PO Box 909, 5600 AX Eindhoven, The Netherlands; 2 Tranzo-Scientific Center for Care and Welfare, Tilburg University, Tilburg, The Netherlands; 3 Curium-LUMC, Child and Adolescent Psychiatry, Leiden University Medical Center, Leiden, The Netherlands; 4 Department of Child and Adolescent Psychiatry, VU University Medical Center, Amsterdam, The Netherlands

**Keywords:** Autism spectrum disorder, Juveniles, Delinquency, Literature overview

## Abstract

**Background:**

Here we present an overview of the literature on autism in adult and juvenile delinquents. We analyzed both the prevalence of autism spectrum disorders (ASD) in groups of delinquents and the prevalence of offending in people with ASD. There is a high prevalence of psychiatric disorders amongst people in custody, but there is disagreement about the prevalence of ASD in this population. Some studies have found overrepresentation of people with ASD in forensic populations whereas others have found that people with ASD have a similar rate of offending to the general population.

**Methods:**

We carried out a systematic search of literature published between 1990 and 2016 and identified studies on the co-occurrence of autism and delinquency using standard search engines.

**Results:**

The prevalence of delinquency in the ASD population varied from 5 to 26%, whilst ASD was found in 2–18% of the forensic populations studied. The reported prevalence of ASD in delinquents and of offending in people with ASD varied widely. This might be due to the use of different diagnostic instruments, the diversity of the samples, the high rate of comorbid psychiatric disorders and the various types of offending behavior.

**Conclusions:**

We cannot conclude from our analysis that people with ASD are more likely to offend than the general population.

## Background

High rates of psychiatric disorders among adolescents in custody have been reported [1–4]. There have been several studies on the prevalence of psychiatric disorders among adolescents in custody, however only a handful have focused specifically on autism spectrum disorders (ASD) and these have produced inconsistent results. The main subtypes of ASD included in this study are autism, Asperger’s syndrome and pervasive developmental disorder. Some have found overrepresentation of people with ASD (particularly Asperger’s syndrome) in forensic settings [5–7] but others have found that the rate of offending is no higher in people with ASD than in the general population [8, 9]. This discrepancy prompted us to produce this overview of the literature on the co-occurrence of autism and delinquency. Delinquency and delinquent behavior are defined as criminal offences. In our paper delinquency is defined as offending behavior; see for instance [10] who defined delinquency as offending behavior with the following different offence types: violent conduct, threatening behavior, property destruction, drug offences, theft, sexual offending, fraud, motoring offences and murder.

Whilst people with ASD generally tend to obey rules, specific symptoms of ASD can predispose individuals to offending behavior; for instance, the abnormal or restricted interests that are typical of ASD can play a role in delinquent behavior [11, 12]. It was suggested that repetitive and stereotyped behaviors were a factor in the exceptional case of the serial sexual homicidal behavior of Jeffrey Dahmer [13]. Schwartz-Watts [14] reported three murder cases in which the ASD symptoms of oversensitivity and difficulty in recognizing facial expressions were seen as relevant. Limited interest, rigidity, and social and communicative problems, which are all symptoms of ASD, may make people with autism more susceptible to delinquent behavior [15, 16]. Impaired ability to understand social information can lead to misinterpretation of others’ intentions and feelings and can, for example, lead to undesirable sexual behavior [17–19]. The role of empathy deficit, as a symptom of ASD, in offending by people with ASD has been described repeatedly in case reports [20–24]. On the other hand, it has also been argued that some symptoms of autism protect people with ASD against involvement in criminal behavior. Many people with Asperger’s syndrome have an overactive sense of right and wrong and are usually conscientious and unwilling to break the law [25].

Several factors not related to specific ASD symptoms may increase the risk of offending in ASD. Several case reports have shown that a late diagnosis of ASD is associated with a higher risk of offending [26–30]. It has also been reported that a lack of appropriate treatment and supervision is a risk factor for violent behavior in patients with ASD [31–33]. In a review pertaining to patients with ASD and the criminal justice system, King and Murphy [34] found that there were some similarities between the difficulties faced by people with ASD and people with intellectual disabilities within the criminal justice system; however, they demonstrated that people with ASD were not overrepresented in the criminal justice system.

Anckarsäter et al. [35] showed that the prevalence of comorbid psychiatric disorders was high in offenders with ASD. Comorbid psychiatric conditions such as psychosis and depression are risk factors for offending behavior in individuals with ASD [36]. It is therefore not surprising that some case reports have illustrated that delinquent behavior in ASD can result from comorbid psychopathology, for example attention deficit hyperactivity disorder and affective disorders [37, 38]. When people with ASD offend it is important to determine whether other psychiatric disorders are also present because it is possible that such conditions influence the risk of offending. A review mainly based on single case reports [39] emphasized the role of psychiatric comorbidity in the association between violent crime and Asperger’s syndrome, noting that 29.7% of the cases included had coexisting psychiatric disorders such as attention deficit hyperactivity disorder (ADHD) and mood disorders.

Until now, most articles and reviews dealing with ASD and offending have been based on case reports. In 1991, Ghaziuddin and colleagues critically evaluated the literature on the incidence of violence in Asperger’s syndrome [8]. The authors analyzed data from a total of 131 patients—15 case reports (covering 23 cases), two case series (covering 37 patients) and four case control studies (covering 71 patients)—and concluded that only three (2.3%) had a clear history of violent behavior. The aims of this study were, therefore, to analyze the prevalence of ASD in delinquent groups and the prevalence of offending behavior in patients with ASD.

## Methods

A computer-assisted search of PsycINFO, PubMed and Embase was conducted to identify all papers about ASD and delinquency published in English between 1990 and 2015. Details of the search strategy can be found in the Appendix. The search terms were deliberately broad, covering a wide range of terms used to refer to ASD and terms for various categories of delinquency. All 6640 abstracts retrieved during the search were screened, and studies related to ASD and delinquency were included. We set no criteria for the age of subjects; publications on both adults and juveniles have been included in our review. Many search terms concerned different terms for ASD, but the search strategy also contained many categories of delinquency, to include all relevant studies.

The initial search was undertaken in 2011 and the same search was repeated every month until the end of 2015. Studies were excluded if they described research on animals, focused primarily on neurobiology or genetics, if subjects had another primary psychiatric illness such as ADHD or a mental handicap, or they pertained to trials of medication or to somatic illnesses. Studies were also excluded if the primary subject of investigation was treatment of ASD, if they considered aggression rather than delinquency and if only infants were studied. The inclusion criteria were publication in English, empirical research, sample of patients with an ASD diagnosis and individuals showing delinquent behavior.

**Fig. 1:**
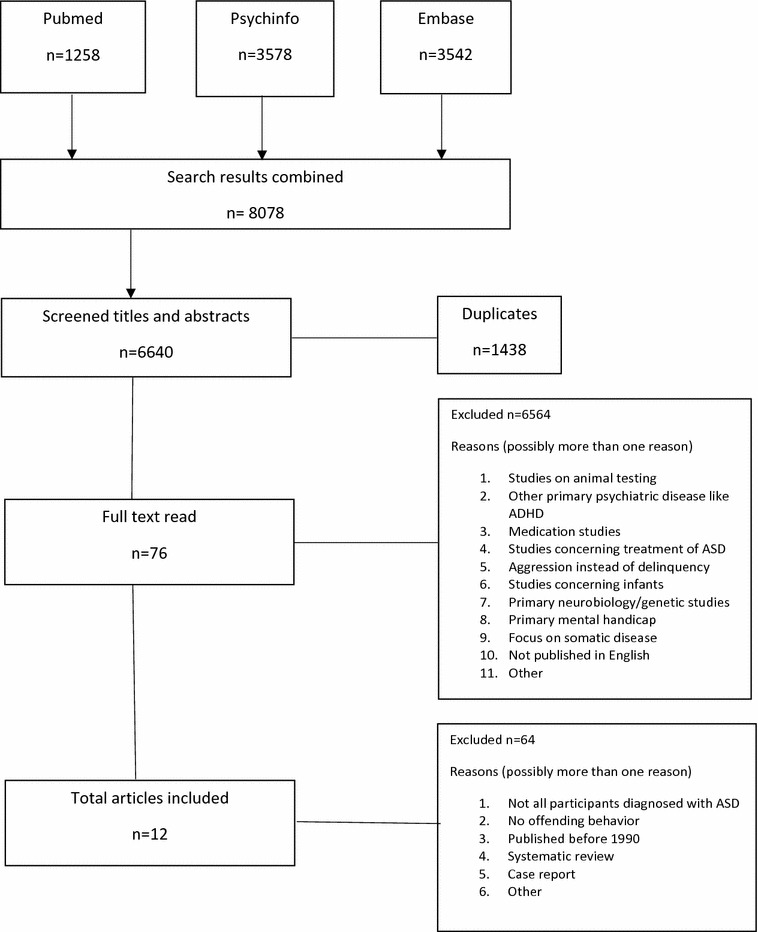
Flow chart of publication selection

All articles that appeared to comply with the selection criteria were reviewed in full (see Fig. 1). The reference lists of the articles were checked in order to identify additional relevant articles.

## Results

### Study selection

The search identified a total of 6640 publications whose titles and abstracts were all checked individually. Based on this check, 6564 abstracts were excluded because they met one or more of the exclusion criteria. Next, the full texts of the 76 potentially eligible articles were critically evaluated. This resulted in the exclusion of a further 64 articles because (a) not all participants were diagnosed with ASD, (b) the study did not deal with offending behavior or (c) the articles were a systematic review or case report. Thus 12 papers were included in this review, five of which report the prevalence of delinquency in patients with ASD and seven the prevalence of ASD in a forensic population.

## Studies of delinquency in ASD

### Sample and study characteristics

These five studies (studies 1–5, Table 1) covered 1672 patients from four different countries: the United Kingdom, Austria, the United States and Denmark. The patients varied in age from 12 to 64 years old and the sample size varied from 25 to 609. One study included juveniles; the other four studies were of adults. The source of data on offending varied from a self-report questionnaire on offending behavior to a juvenile justice database and penal and criminal register.

**Table 1:** Studies of prevalence of delinquency in patients with autism spectrum disorders

Authors	*N*	Setting	Diagnosis and classification system	Type of instrument/source of data on offending	Age in years	Control group	Conclusion	Type of delinquency
Allen et al. [10]	33 of 126; 26%	Mostly mental health services but also probation services and prisons	Asperger’s syndrome classification system unknown	Questionnaire covering offending behavior + semi-structured interview	18–61; *M* = 34.8	None	No association between Asperger’s syndrome and offending	Violent behavior and threatening conduct most common followed by destructive behavior, drug offenses and theft
Woodbury-Smith et al. [41]	2 of 25; 8%	Primary care services, mental health services, learning disability services and local media	High-functioning autism/Asperger’s syndrome ICD-10	Self-Reported Offending Questionnaire and Home Office(UK) Offenders Index	*M* = 29.8	20 non-ASD comparison group	Rating of offending lower in the ASD groups than in the non-ASD comparison group	More criminal damage in ASD group and fewer drug offenses in ASD group
Hippler et al. [40]	33 in 177; 19%	Vienna University Children’s clinic and institute for mental history	Autistic psychopathy and Asperger’s syndrome ICD-10	Criminal records search of the Austrian Penal Register	23–64; *M* = 42	None	Asperger’s patients no more likely to have been convicted of a crime than the general male population	Most common conviction in Asperger patients property offenses and second falsification or suppression of documents
Mouridsen et al. [9]	29 in 313; 9%	University Clinics of Child Psychiatry of Copenhagen and Aarhus	13 childhood autism, 86 atypical autism and 114 Asperger’s syndrome ICD-9, ICD-10	Danish Criminal Register	*M* = 24.5	933 matched controls	Offenders with atypical autism and Asperger’s convicted of all kinds of offenses	Significantly more arson in Asperger patients and fewer violations for traffic law
Cheely et al. [42]	32 of 609; 5%	Department of juvenile justice, South Carolina law enforcement division and South Carolina autism and developmental disabilities monitoring program	Autism spectrum disorder DSM-IV-TR	Department of Juvenile Justice and South Carolina Law Enforcement Division databases	12–18	99 matched controls	Youths with ASD had lower rates of charges overall	Higher rate of charges of offenses against the person in youths with ASD; lower rate of charges of property offenses and fewer charges with probation violations

### Prevalence of delinquency in ASD

As can be seen in Table 1, the prevalence of offending behavior varied substantially in people with ASD. Just over a quarter of a sample of people with Asperger’s syndrome (33 out of 126; 26%) had engaged in offending behavior [10] and a similar result was found in an Austrian cohort, in which 33 out of 177 patients (19%) had offended [40]. In a study of 25 high-functioning patients with autism or Asperger’s syndrome the rate of offending was even lower (8%) [41]. This rate is comparable with the low rates observed in other studies of people with ASD: 5% (32/609) [42] and 9% (29/303) [9].

Three studies have compared the prevalence of various criminal offences amongst people with ASD and the general population. A Danish study [9] that compared 313 ASD patients with 933 matched controls found that people with Asperger’s syndrome were less likely to have committed traffic offences than matched controls, but more likely to have committed arson. In the Austrian cohort [40], most convictions were for property offences (81% of all convictions) whilst offences against life and physical integrity were rare (9%). In the study of Cheely and colleagues the rate of crimes against the person was higher in juveniles with ASD than in matched controls, although the rate of property crimes was lower. In this study the juvenile offenders with ASD were less likely to have a comorbid intellectual disability than the general juvenile population with ASD in South Carolina [42].

Comorbidity has been described in people with ASD who offend. Sixteen out of 33 offenders with Asperger’s syndrome [10] had various additional psychiatric diagnoses, most commonly schizophrenia (25%), followed by ADHD (18.75%), depression (12.5%), and anxiety disorder and personality disorder (both 6.25%).

## Studies of ASD in delinquents

### Sample and study characteristics

The seven selected studies (studies 6–12, Table 2) covered 4107 offenders from four different countries: Sweden, the Netherlands, Japan and the United Kingdom. The sample size varied from 69 to 2395 and the mean age from 10.7 to 34.4 years old. One study did not report offenders’ ages [43]. Two studies had mixed adult and juvenile samples, two used adult-only samples and three were limited to juveniles.

**Table 2:** Studies of prevalence of autism spectrum disorders in suspected and delinquent populations

	Results	Setting	Diagnosis and classification system	Type of instrument used to diagnose autism	Age in years	Control group	Conclusion
Scragg and Shah [43]	ASD prevalence: 2.3% in 392 patients held in Broadmoor secure hospital	Secure hospital	Asperger’s syndrome Gillberg and Gillberg criteria	Examination, Screening Schedule for Autistic Behavior and interview	Not reported	None	Prevalence of Asperger’s syndrome in Broadmoor Hospital higher than reported for general population
Anckarsäter et al. [35]	ASD prevalence = 13% in 3 Swedish cohorts (*n* = 100, *n* = 100, *n* = 130)	Special hospital for forensic psychiatry, violent or sexual offenders who were undergoing pre-trial investigation at department of forensic psychiatry and institutions of maladapted youths	Autism, Asperger’s syndrome and atypical autism Gillberg and Gillberg criteria and DSM-IV	Clinical examinations, SCID-I, ASDI, ASSQ	Group 1: *M* = 27; group 2: *M* = 25.5; group 3: *M* = 15	None	ASD a clinically relevant problem among forensic populations
Enayati et al. [45]	Prevalence of Asperger’s syndrome: 7.1% in 214 arsonists; 2.5% in 2395 other violent offenders	Convicted offenders	Asperger’s syndrome DSM-IV	None; Forensic psychiatric investigations	*M* = 34.4	2395 other violent offenders	Male arsonists compared with other violent offenders more often diagnosed with Asperger’s syndrome
Geluk et al. [44]	Incidence rate ratio 1.29; (total symptom score) in 308 first-time child arrestees	Childhood arrestees by the police	Autistic symptoms conform DSM-IV-TR	Children’s Social Behavior Questionnaire	*M* = 10.7	840 matched controls	Autistic symptoms predict future delinquent behavior in childhood arrestees
‘t Hart-Kerkhoff et al. [18]	Higher level of ASD symptoms in 175 suspected juvenile sex offenders compared with matched controls	Juvenile suspected sex offenders	ASD symptoms conform DSM-IV-TR	Children’s Social Behavior Questionnaire	Offenders: *M* = 14.9; ASD: *M* = 14.2	500 matched healthy controls, *M* age 14.0 years	Level of ASD symptoms higher in juvenile sex offenders, especially solo offenders and child molesters, than in group offenders
Kumagami and Matsuura [46]	In 428 family court juvenile cases a pervasive developmental disorder prevalence of 3.2–18.2%	Family court juvenile cases	Pervasive developmental disorder (PDD) DSM-IV	Diagnosing and subtyping of PDD and type of crime by interview and school and court records	*M* = 17	None	In PDD group significantly higher rate of sex-related crimes than in other juveniles referred to family courts
Siponmaa et al. [7]	ASD prevalence: 15% in young offenders referred for forensic psychiatric investigation		Pervasive developmental disorder and Asperger’s syndrome ICD-10, DSM-IV, Gillberg and Gillberg criteria	Semi-structured psychiatric interview and psychiatric state examination	Range 15–22	None	High prevalence of ASD in young offenders referred for forensic psychiatric investigation

The type of instrument used to diagnose ASD or detect symptoms of ASD varied, from self-report questionnaires and a questionnaire measuring autistic symptoms to forensic psychiatric examination. Two studies used a parent-report instrument, the Children’s Social Behavior Questionnaire (CSBQ) [18, 44]. One study used two questionnaires specifically designed to detect Asperger’s syndrome, the Asperger’s Syndrome Diagnostic Interview (ASDI) and the Asperger’s Syndrome Screening Questionnaire (ASSQ) [35]. One study used the Screening Schedule for Autistic Behavior [43] and in three studies ASD was diagnosed by psychiatric examination [7, 35, 45].

### Prevalence of ASD in delinquents

Table 2 shows that the prevalence of ASD in the suspected and delinquent populations varied from 2.3% [43] to 15% [7]. Different categories of delinquency and specific offender groups such as very young offenders have been studied. The prevalence of Asperger’s syndrome in the male population of Broadmoor high-security hospital was the lowest reported in all the studies of offender populations at 2.3%, but this is still higher than in the general population [43]. A retrospective study of the prevalence of child neuropsychiatric disorders amongst adolescent offenders (15–22 years old) referred for psychiatric investigation reported an ASD prevalence of 15% [7]. In a sample of 428 juvenile cases heard in the family court, the prevalence of pervasive developmental disorder among the offenders ranged from 3.2 to 18.2% depending on the nature of the offence; it was higher amongst those charged with sex crimes and lower in those charged with property crimes [46].

Two studies investigated the prevalence of ASD in a specific category of offender. A Swedish study [45] compared arsonists with other violent offenders referred for forensic psychiatric assessment; Asperger’s syndrome was diagnosed more often in the arsonist group (7.1%) than in the nonarsonist group (2.5%). A Dutch study of juvenile sex offenders showed that compared with group sex offenders, solo peer sex offenders and child molesters had higher total CSBQ scores and higher scores on several subscales [18].

A Dutch study compared the prevalence of autistic symptoms in very young (baseline age 10.7 years) first-time arrestees with the prevalence in the general population and in children with ASD [44]. Symptoms were measured at baseline and 1 year later. The young offenders had higher total CSBQ scores, higher core symptom scores and higher scores on all CSBQ subscales than the general population, but their scores were lower than those of the group of children with ASD. In childhood arrestees autistic symptoms were positively associated with delinquent behavior.

A study of the prevalence and specific features of ASD amongst individuals in a forensic psychiatric hospital, a department of forensic psychiatry and special youth centers reported an ASD prevalence of 13% based on clinical examinations and the ASDI (Asperger Syndrome Diagnostic Interview) [35]. In most cases the diagnosis was supported by the Asperger Syndrome Screening Questionnaire (ASSQ) results, and in the forensic psychiatry group by the Structured Clinical Interview for DSM-IV axis I (SCID-I) as well. The incidence of comorbidity was remarkably high, 81–100%, and included diagnoses of ADHD, affective illnesses, psychotic disorders, substance use disorders and personality disorders.

## Discussion

The aim of this article was to present an overview of the literature on the co-occurrence of autism and delinquency in adults and juveniles. We have reviewed both research focusing on delinquency in people with ASD and research on the prevalence of ASD in forensic populations. The studies included in our review suggest ASD and autistic symptoms are more prevalent in forensic populations. With regard to the results, this article shows that the prevalence of ASD in forensic populations varied from 2.3% [43] to 15% [7], which is higher than in the general population. In contrast, the rate of offending was lower in people with ASD than in the general population, ranging from 5% [42] to 26% [10], which is still not higher than in the general population.

Overall, the variance in prevalence was high, probably due to variation in the instruments used to diagnose ASD and to the diversity of the samples studied. ASD was much more prevalent in young offenders referred for forensic psychiatric investigation (15%; [7] than in patients in a secure hospital (2.3%; [43]. Furthermore, a high rate of comorbidity was observed [35]. This finding is in accordance with a review based mainly on case reports, in which only 6 out of 37 violent offenders with Asperger’s syndrome had no additional psychiatric disorder [39]. It is likely that in this group, comorbid mental disorders had increased the risk of offending behavior and therefore patients with Asperger’s syndrome who have committed a crime should be assessed for comorbid psychiatric disorders. In the case of comorbidity it is difficult to determine whether ASD or the comorbid psychiatric disorder affects the risk of offending behavior.

The prevalence has been studied from a different starting point: the prevalence of ASD in suspected and delinquent groups and the prevalence of delinquency in people with ASD. All studies of delinquent groups reported a higher prevalence of ASD than in the general population, where it is 0.3–0.6% [47]. The prevalence of ASD or symptoms of ASD in the suspected and delinquent populations varied between 2.0 and 15.0%. It is not surprising that people with ASD are overrepresented in this population of delinquents and people who have been accused of committing a crime; two of the seven studies used a sample drawn from patients in a forensic psychiatric hospital [35, 43] and two studied ASD in offenders referred for forensic psychiatric assessment [7, 45], thereby increasing the probability that subjects would have a psychiatric diagnosis, including ASD. In the two Dutch studies, symptoms of ASD were assessed using parent-reported CSBQ data. Because of these limitations, the nature of the forensic sample in four of the selected studies and the use of a measure of autistic symptoms rather than a diagnosis of ASD in two studies, the actual prevalence of ASD in forensic populations might be different from the figures reported here. Both a clinical examination and a heteroanamnesis are required to diagnose ASD. Validated diagnostic instruments should be used whenever possible, but validated diagnostic instruments such as the Autism Diagnostic Observation Schedule (ADOS) and Autism Diagnostic Interview (ADI) were not used in any of the studies included in this review. Of the instruments used to diagnose ASD, the ADI and ADOS have the largest evidence base and highest sensitivity and specificity [48]. A disadvantage to using these instruments is that they are time-consuming to administer and cannot replace a clinical examination. It is possible that some people with ASD have low ADOS and ADI scores and vice versa.

Contrary to our expectations, the prevalence of delinquency was lower in all the samples of people with ASD than in the general population. In the general juvenile population, the self-reported prevalence of delinquency is 45.0% [49]. Variance in the methods used to assess offending, which ranged from criminal registers to self-reported questionnaires, undoubtedly contributed to this variation, but heterogeneity in the ASD samples may also be relevant.

The prevalence of ASD diagnoses, particularly Asperger’s syndrome, in forensic settings is remarkable because it is much higher than the prevalence of ASD diagnoses in the general population. One study found that arsonists were more likely than other violent offenders referred for forensic examinations to be diagnosed with Asperger’s syndrome [45]. A Dutch study of juvenile sex offenders showed that solo peer sex offenders and child molesters in particular had high total CSBQ scores and higher scores on several subscales [18]. On the other hand, people with ASD appear to be no more likely to offend than the general population, perhaps because many people with ASD have an overactive sense of right and wrong and are usually conscientious and unwilling to break the law [25].

Some symptoms of autism, such as the overactive sense of right and wrong and the unwillingness to break the law, tend to protect people with ASD from committing criminal behavior. Other symptoms of ASD, such as a tendency to misread the behavior of others, constitute a risk factor for offending behavior. There are studies that show that the diagnosis of ASD is more prevalent amongst those who have committed some categories of offence, for example some sex offences, than for other psychiatric diagnoses whereas ASD is less prevalent amongst offenders convicted of other categories of offence, such as property crimes. Comparing the studies in this overview is unfortunately difficult as different instruments have been used to indicate offending. Some studies used criminal records or registers whereas others relied on self-report questionnaires or interviews, and it has been established that the self-reported prevalence of offending is much higher than the official crime rate, especially at younger ages [49].

### Limitations

First, this review covers only a limited number of studies; whilst there have been many case reports, the number of prevalence studies is much smaller. Although many researchers have suggested that there is an association between ASD and delinquency, only 12 prevalence studies met the selection criteria for this review.

The included studies are from a diverse group of countries with different judicial systems, methods of diagnosing ASD and instruments for assessing symptoms of ASD. This makes it difficult to compare them.

There are only a small number of prevalence studies of delinquency in juvenile patients with ASD. In the studies of the prevalence of ASD in suspected and delinquent populations there are many more studies concerning juveniles.

## Conclusions and implications for further research

The relationship between ASD and delinquency is complex. The extant research shows that for most people with ASD there is no association between ASD and delinquent behavior. Although the nature of the relationship between ASD and delinquency is not clear, it is clear that it is affected by factors such as comorbidity, specific symptoms of ASD and the type of crime.

It would be useful to investigate the prevalence of ASD in different offender categories. It would also be interesting to find out whether some people with ASD are only diagnosed when they commit a crime. It is possible that there is a tendency to diagnose ASD more often in people who have committed specific types of crime and this is an area that warrants more extensive research. It is important to diagnose ASD carefully and to differentiate autism symptoms such as a lack of empathy from psychopathic traits, and this can sometimes be difficult. Earlier diagnosis should ensure that people with ASD receive better care and may help to prevent them offending.

## Appendix

### PsycINFO

(DE “Pervasive Developmental Disorders” OR DE “Aspergers Syndrome” OR DE “Autism” OR DE “Rett Syndrome” OR DE “Autistic Thinking” OR TX Autis* OR TX Asperger* OR TX PDD OR (Pervasi* W3 Disorder*)) AND ((DE “Criminals” OR DE “Crime” OR DE “Criminal Behavior” OR DE “Violent Crime” or DE “Serial Crime” OR DE “Perpetrators” OR DE “Female Criminals” OR DE “Male Criminals” OR DE “Mentally Ill Offenders” OR DE “Perpetrators” OR DE “Juvenile Delinquency” OR DE Predelinquent Youth OR DE “Female Delinquency” OR DE “Male Delinquency” OR DE “Aggressive Behavior” OR DE “Aggressive Driving Behavior” OR DE “Animal Aggressive Behavior” OR DE “Attack Behavior” OR DE “Coercion” OR DE “Aggressiveness” OR DE “Driving Under the Influence” OR DE “Hate Crimes” OR DE “Human Trafficking” OR DE “Illegal Drug Distribution” OR DE “Kidnapping” OR DE “Serial Crime” OR DE “Vandalism” OR DE “Violence” OR DE “Cruelty” OR DE “Torture” OR DE “Intimate Partner Violence” OR DE “Patient Violence” OR DE “Elder Abuse” OR DE “Emotional Abuse” OR DE “Harassment” OR DE “Partner Abuse” OR DE “Child Neglect” OR DE “Battered Child Syndrome” OR DE “Domestic Violence” OR DE “Physical Abuse” OR DE “Patient Abuse” OR DE “Persecution” OR DE “Terrorism” OR DE “Verbal Abuse” OR DE “School Violence” OR DE “Workplace Violence” OR DE “Political Assassination” OR DE “Terrorism” OR TX Crime* OR TX Criminal* OR TX Criminol* OR TX Delinquen* OR TX Misdemeanor* OR TX Felonies OR TX Perpetrator* OR TX Offender* OR TX Offens* OR TX Aggressi* OR TX Violen* OR TX Assault* OR (TX Agnostic AND (TX Behavior OR TX Behaviour)) OR TX Abduct* OR TX Kidnap* OR TX Delinquen* OR ZK “criminal behavior & juvenile delinquency” OR CC 3236) OR (DE Recidivism OR TX Recidivis* OR TX Relaps* OR TX Recrude* OR TX Reoffend* OR (TX Repeat* AND (TX Offen* OR TX Delinquen* OR TX Crime* OR TX Criminal* OR TX Criminol*))) OR (DE Psychopathy OR DE Antisocial Behavior OR DE Antisocial Personality Disorder OR TX Psychopath OR TX Psychopaths OR TX Psychopathy OR TX Psychopathic OR TX Sociopath* OR TX ASPD OR ((TX Antisocial* OR TX Dissocial*) AND (TX Person* OR TX Behavior* OR TX Behaviour*))) OR (DE “Sex Offenses” OR DE “Stalking” OR TX Stalk* OR DE “Sexual Abuse” OR DE “Rape” OR DE “Acquaintance Rape” OR DE Incest OR DE Pedophilia OR (TX Sex* AND (TX Offen* OR TX Crime* OR TX Criminal* OR TX Criminol* OR TX Delinquen* OR TX Abus* OR TX Aggress* OR TX Violen* OR TX Assault* OR TX Murder* OR TX Homicid* OR TX Perpetrat* OR TX Harras*)) OR TX Rape OR TX Raping* OR TX Rapist* OR TX Incest OR TX Paedophil* OR TX Pedophil* OR (TX Child* AND TX Molest*)) OR (DE Pedophilia OR DE “Child Abuse” OR TX Pedoph* OR TX Pedosex* OR TX Paedophil* OR (TX Rape* OR TX Rapist* OR (TX Sex* AND (TX Abus* OR TX Offend* OR TX Molest*))) AND (TX Kids OR TX Kid OR TX Child*)) OR (DE “Theft” OR DE “Shoplifting” OR DE Kleptomania OR TX Theft* OR TX Kleptoman* OR TX Thief OR TX Thieves OR TX Shoplift* OR TX Robber* OR TX Stealing OR TX Burglar*) OR (DE Pyromania OR TX Pyroman* OR DE Arson OR TX Arson* OR TX Firesett* OR TX incendiary* OR (TX Fire* AND TX Set*)) OR (DE “Homicide” OR DE “Serial Homicide” OR DE Filicide OR DE Infanticide OR TX Homicid* OR TX Murder* OR TX Manslaught* OR TX Uxoricid* OR TX Parricid* OR TX Matricid* OR TX Familicid* OR TX Patricid* OR TX Siblicid* OR TX Filicid* OR TX Femicid* OR TX Parricid* OR TX Infanticid* OR TX Neonaticid* OR (TX Violen* AND (TX Death OR TX Lethal)) OR (TX Child* AND (TX Homicid* OR TX Kill* OR TX Murder*)) OR TX Kill* OR ((TX Serial OR TX Multiple OR TX Mass) AND (TX Homicid* OR TX Kill* OR TX Murder*)) OR TX Assassinat*) OR (TX Neonaticid* OR ((TX Murder* OR TX Kill* OR TX Homicid* OR TX Infanticid*) AND (TX Newborn* OR TX Baby OR TX Babies OR TX Neonat*))) OR (DE “Penology” OR DE “Forensic Psychiatry” or DE “Criminal Justice” OR DE “Criminal Conviction” OR DE “Juvenile Justice” or DE “Forensic Evaluation” or DE “Forensic Psychology” OR DE Criminology OR ((TX Crime* OR TX Criminal* OR TX Criminol* OR TX Penal*) AND (TX Justice OR TX Convict* OR TX Law)) OR ((TX Forensic OR TX Legal) AND (TX psychiatr* OR TX psycholog* OR TX Evaluat* OR TX Health* OR TX Care OR TX Nurs*)) OR TX Penolog* OR ZK “criminal law & adjudication” OR ZK “criminal rehabilitation & penology” OR ZK “forensic psychology & legal issues” OR CC 3236 OR CC 3386 OR CC 4200 OR CC 4230 OR CC 4270) OR (DE Prisons OR DE Prisoners OR DE Incarceration OR DE Probation OR DE Correctional Institutions OR DE Legal Detention OR TX Prison* OR TX Imprison* OR TX Jail* OR TX Inmat* OR TX Penitent* OR TX Custod* OR TX Detention* OR TX Detain* OR TX Probati* OR TX Incarcerat* OR TX Gaol* OR ((TX Penal* OR TX Correct*) AND (TX Institut* OR TX System*)))) AND ((AG “adolescence (13-17 yrs)” OR AG “childhood (birth-12 yrs)” OR AG “preschool age (2-5 yrs)” OR AG “school age (6-12 yrs)” OR AG “young adulthood (18-29 yrs)” OR TX Youth* OR Youngster* OR TX Juvenil* OR TX Teen* OR TX Adolescen* OR TX Puberty OR TX Preschool* OR TX Child* OR (Young N3 Adult*) OR ((TX Preschool OR TX School) AND TX Age)) OR (DE “Juvenile Delinquency” OR DE Predelinquent Youth OR DE Juvenile justice OR CC 3236)) AND (PY 1990-2015)

3578 hits

### PubMed

(Child Development Disorders, Pervasive[MeSH] OR (Pervasi*[tiab] AND Disorder*[tiab]) OR Autis*[tiab] OR Asperger*[tiab] OR (“Theory of Mind”[tiab])) AND ((Aggression[MeSH] OR Violence[MeSH] OR Crime[MeSH] OR Criminal Psychology[MeSH] OR Juvenile Delinquency[MeSH] OR Crime*[tiab] OR Criminal*[tiab] OR Criminol*[tiab] OR Delinquen*[tiab] OR Misdemeanor*[tiab] OR Felonies[tiab] OR Perpetrator*[tiab] OR Offender*[tiab] OR Offens*[tiab] OR Aggressi*[tiab] OR (Agnostic[tiab] AND (Behavior[tiab] OR Behaviour[tiab]))OR Violen*[tiab] OR Assault*[tiab] OR Delinquen*[tiab] OR Abduct*[tiab] OR Kidnap*[tiab]) OR (Recidivis*[tiab] OR Reoffend*[tiab] OR ((Repeat*[tiab] OR Relaps*[tiab] OR Recrude*[tiab]) AND (Offen*[tiab] OR Crime[MeSH] OR Juvenile Delinquency[MeSH] OR Crime*[tiab] OR Criminal*[tiab] OR Criminol*[tiab] OR Violen*[tiab] OR Delinquen*[tiab] OR Violence[Mesh]))) OR (Antisocial Personality Disorder[MeSH] OR Psychopath[tiab] OR Psychopaths[tiab] OR Psychopathy[tiab] OR Psychopathic[tiab] OR Sociopath*[tiab] OR ASPD[tiab] OR ((Antisocial*[tiab] OR Dissocial*[tiab]) AND (Person*[tiab] OR Behavior*[tiab] OR Behaviour*[tiab]))) OR (Sex Offenses[MeSH] OR Sexual Harassment[MeSH] OR Stalking[MeSH] OR Incest[MeSH] OR Pedophilia[MeSH] OR (Sex*[tiab] AND (Offen*[tiab] OR Crime*[tiab] OR Criminal*[tiab] OR Criminol*[tiab] OR Delinquen*[tiab] OR Abus*[tiab] OR Aggress*[tiab] OR Violen*[tiab] OR Assault*[tiab] OR Murder*[tiab] OR Homicid*[tiab] OR Perpetrat*[tiab] OR Harras*[tiab])) OR Stalk*[tiab] OR Rape[tiab] OR Raping*[tiab] OR Rapist*[tiab] OR Incest[tiab]) OR (Pedophilia[MeSH] OR Child Abuse[MeSH] OR Child Abuse, Sexual[MeSH] OR Pedoph*[tiab] OR Pedosex*[tiab] OR Paedophil*[tiab] OR (Rape*[tiab] OR Rapist*[tiab] OR (Sex*[tiab] AND (Abus*[tiab] OR Offend*[tiab] OR Molest*[tiab]))) AND (Kids[tiab] OR Kid[tiab] OR Child*[tiab])) OR (Theft[MeSH] OR Theft*[tiab] OR Kleptoman*[tiab] OR Thief[tiab] OR Thieves[tiab] OR Shoplift*[tiab] OR Robber*[tiab] OR Stealing[tiab] OR Burglar*[tiab]) OR (Firesetting Behavior[MeSH] OR Pyroman*[tiab] OR Arson*[tiab] OR Firestart*[tiab] OR Firesett*[tiab] OR incendiar*[tiab] OR (Fire*[tiab] AND Set*[tiab])) OR (Homicide[MeSH] OR Infanticide[MeSH] OR Homicid*[tiab] OR Murder*[tiab] OR Manslaught*[tiab] OR Filicid*[tiab] OR Femicid*[tiab] OR Parricid*[tiab] OR Uxoricid*[tiab] OR Parricid*[tiab] OR Matricid*[tiab] OR Familicid*[tiab] OR Patricid*[tiab] OR Siblicid*[tiab] OR Neonaticid*[tiab] OR (Violen*[tiab] AND (Death[tiab] OR Lethal[tiab])) OR Infanticid*[tiab] OR (Child*[tiab] AND (Homicid*[tiab] OR Kill*[tiab] OR Murder*[tiab])) OR ((Serial[tiab] OR Multiple[tiab] OR Mass[tiab]) AND (Homicid*[tiab] OR Kill*[tiab] OR Murder*[tiab])) OR Assassinat*[tiab]) OR (Neonaticid*[tiab] OR ((Murder*[tiab] OR Homicid*[tiab]) AND (Newborn*[tiab] OR Baby[tiab] OR Babies[tiab] OR Neonat*[tiab]))) OR (Infant, Newborn[MeSH] AND (Homicide[MeSH] OR Infanticide[MeSH] OR Homicid*[tiab] OR Murder*[tiab] OR Infanticid*[tiab])) OR (Forensic Psychiatry[MeSH] OR Criminal Law[MeSH] OR Criminology[MeSH] OR ((Crime*[tiab] OR Criminal*[tiab] OR Criminol*[tiab] OR Penal*[tiab]) AND (Justice[tiab] OR Convict*[tiab] OR Law[tiab])) OR ((Forensic[tiab] OR Legal[tiab]) AND (Psychiatr*[tiab] OR Psycholog*[tiab] OR Evaluat*[tiab] OR Health*[tiab] OR Care[tiab] OR Nursing[tiab])) OR Penolog*[tiab]) OR (Prisons[MeSH] OR Prisoners[MeSH] OR Incarcerat*[tiab] OR Probati*[tiab] OR Prison*[tiab] OR Imprison*[tiab] OR Jail*[tiab] OR Inmat*[tiab] OR Penitent*[tiab] OR Custod*[tiab] OR Detention*[tiab] OR Detain*[tiab] OR Probati*[tiab] OR Incarcerat*[tiab] OR Gaol*[tiab] OR ((Penal*[tiab] OR Correct*[tiab]) AND (Institut*[tiab] OR System*[tiab])))) AND (Adolescent[MeSH] OR Young Adult[MeSH] OR Child[MeSH] OR Infant[MeSH] OR Child[All Fields] OR Children[tiab] OR Adolescen*[tiab] OR Puberty[tiab] OR Youth*[tiab] OR Young*[tiab] OR Juvenil*[tiab] OR Toddler*[tiab] OR Infan*[tiab] OR Boy*[tiab] OR Girl*[tiab] OR Preschool*[tiab] OR (School[tiab] AND Age[tiab])) AND (“1990”[PDAT] : “2015”[PDAT])

1258 hits

### Embase

(exp *Autism/ OR ((Pervasi* AND Disorder*) OR Autis* OR Asperger* OR ASD OR (Theory of Mind)).mp) AND (((exp Crime/ OR exp Offender/ OR exp Delinquency/ OR exp Juvenile Delinquency/ OR exp Aggression/ OR exp Violence/) OR (Crim* OR Delinquen* OR Misdemeanor* OR Felonies OR Perpetrator* OR Offend* OR Offens* OR Aggressi* OR Agnostic OR Violen* OR Assault* OR Delinquen* OR Abduct* OR Kidnap*).mp) OR (exp Recidivism/ OR Reoffend*.mp OR ((Repeat* OR Relaps* OR Recurren* OR Recrude*).mp AND (exp Crime/ OR exp Delinquency/ OR exp Juvenile Delinquency/ OR exp Violence/ OR (Crim*.mp OR Violen* OR Offen* OR Delinquen*).mp))) OR (exp Psychopathy/ OR Antisocial behavior/ OR Sociopathy/ OR (Psychopath OR Psychopaths OR Psychopathy OR Psychopathic OR Sociopath* OR ASPD OR ((Antisocial* OR Dissocial*) AND (Person* OR Behavior* OR Behaviour*))).mp) OR (exp Sexual Crime/ OR exp Rape/ OR exp Sexual abuse/ OR exp Incest/ OR exp Stalking/ OR ((Sex* AND (Offen* OR Crim* OR Delinquen* OR Abus* OR Aggress* OR Violen* OR Assault* OR Murder* OR Homicid* OR Perpetrat* OR Harras*)) OR Rape OR Raping* OR Rapist* OR Stalk* OR Incest).mp) OR (exp Pedophilia/ OR exp Child abuse/ OR exp Child Sexual Abuse/ OR (Pedoph* OR Pedosex* OR Paedophil* OR (Rape* OR Rapist* OR (Sex* AND (Abus* OR Offend* OR Molest*))) AND (Kids OR Kid OR Child*)).mp) OR (exp Theft/ OR exp Kleptomania/ OR (Theft* OR Kleptoman* OR Thief OR Thieves OR Shoplift* OR Robber* OR Stealing OR Burglar*).mp) OR (exp Arson/ OR exp Pyromania/ OR (Pyroman* OR Arson* OR Firesett* OR Firestart* OR incendiar* OR (Fire* AND Set*)).mp) OR (exp Homicide/ OR exp Infanticide/ OR (Homicid* OR Murder* OR Kill* OR Manslaught* OR Filicid* OR Femicid* OR Uxoricid* OR Parricid* OR Matricid* OR Familicid* OR Patricid* OR Siblicid* OR Neonaticid* OR Infanticid* OR Assassinat* OR (Violen* AND (Death OR Lethal)) OR ((Child* OR Kids OR Kid) AND (Homicid* OR Kill* OR Murder*)) OR ((Serial OR Multiple OR Mass) AND (Homicid* OR Kill* OR Murder*))).mp) OR ((Neonaticid* OR ((Murder* OR Homicid* OR Infanticid*) AND (Newborn* OR Baby OR Babies OR Neonat*))).mp) OR (exp *Newborn/ AND (exp Homicide/ OR exp Infanticide/ OR (Murder* OR Homicid* OR Infanticid*).mp)) OR (exp Forensic Psychiatry/ OR exp Criminal Law/ OR exp Criminology/ OR (((Crim* OR Penal*) AND (Justice OR Convict* OR Law)) OR ((Forensic* OR Legal*) AND (Psychiatr* OR Psycholog* OR Evaluat* OR Health* OR Care OR Nurs*)) OR Penolog*).mp) OR (exp Prisons/ OR exp Prisoners/ OR (Prison* OR Imprison* OR Jail* OR Inmat* OR Penitent* OR Custod* OR Detention* OR Detain* OR Probati* OR Incarcerat* OR Gaol* OR ((Penal* OR Correct*) AND (Institut* OR System*))).mp)) AND (Child* OR Adolescen* OR Puberty OR Youth* OR Young* OR Juvenil* OR Infan* OR Toddler* OR Infan* OR Boy* OR Girl* OR Preschool* OR (School AND Age)).mp AND (“1990” or “1991” or “1992” or “1993” OR “1994” or “1995” or “1996” or “1997” or “1998” or “1999” or “2000” or “2001” or “2002” or “2003” or “2004” or “2005” or “2006” or “2007” or “2008” or “2009” or “2010” or “2011” or “2012” or “2013” or “2014” or “2015”).yr.

3242 hits

## Abbreviations

ASD: autism spectrum disorder
CSBQ: Children’s Social Behavior Questionnaire
